# Generation and Characterization of Site‐Specifically Mono‐Ubiquitylated p53

**DOI:** 10.1002/cbic.202100659

**Published:** 2022-02-03

**Authors:** Alexandra Julier, Vanessa Radtke, Andreas Marx, Martin Scheffner

**Affiliations:** ^1^ Departments of Biology and Chemistry Konstanz Research School Chemical Biology University of Konstanz Universitätsstr. 10 78457 Konstanz Germany

**Keywords:** bioconjugation, oxime ligation, protein modifications, tumor suppressor p53, ubiquitylation

## Abstract

The tumor suppressor p53 is regulated by various posttranslational modifications including different types of ubiquitylation, which exert distinct effects on p53. While modification by ubiquitin chains targets p53 for degradation, attachment of single ubiquitin moieties (mono‐ubiquitylation) affects the intracellular location of p53 and/or its interaction with chromatin. However, how this is achieved at the molecular level remains largely unknown. Similarly, since p53 can be ubiquitylated at different lysine residues, it remains unclear if the eventual effect depends on the position of the lysine modified. Here, we combined genetic code expansion with oxime ligation to generate p53 site‐specifically mono‐ubiquitylated at position 120. We found that mono‐ubiquitylation at this position neither interferes with p53 ubiquitylation by the E3 ligases HDM2 and E6AP in complex with the viral E6 oncoprotein nor affects p53 binding to a cognate DNA sequence. Thus, ubiquitylation per se does not affect physiologically relevant properties of p53.

## Graphical abstract

**The ubiquitin (Ub)** system (E1, E2, E3) ubiquitylates the tumor suppressor p53 thereby affecting different p53 properties including interaction with DNA and other proteins. However, as p53 is ubiquitylated at several lysine residues, it remains unclear, if and how distinct p53 properties are affected by site‐specific ubiquitylation. To address this pertinent question, oxime ligation was employed to generate site‐specifically mono‐ubiquitylated p53.

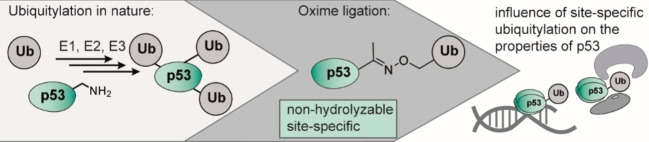

The growth‐suppressive properties of the tumor suppressor p53 are negatively affected in the majority of human cancers by different means including mutations in the TP53 gene^[1]^ or overexpression of the proto‐oncoprotein HDM2, a member of the RING family of E3 ubiquitin ligases.^[2]^ p53 is best known for its ability to act as a sequence‐specific transcriptional modulator that upon various stress stimuli, is activated and in consequence modulates the expression of various genes.^[3]^ Thereby, p53 triggers different cellular responses including cell cycle arrest, apoptosis, senescence, alteration of metabolic pathways, and autophagy.^[3]^ To control p53 function properly under normal growth conditions and upon stress, p53 is subject to various posttranslational modifications (PTMs) including phosphorylation, acetylation, and ubiquitylation, to name but a few.^[4]^ Ubiquitylation is a highly complex and versatile PTM that ranges from the attachment of a single ubiquitin (Ub) moiety to a substrate protein (mono‐ubiquitylation) to the attachment of a sheer indefinite number of differently linked Ub chains (poly‐ubiquitylation).^[5]^ p53 is subject to both, mono‐ and poly‐ubiquitylation. HDM2‐mediated poly‐ubiquitylation, for instance, targets p53 for degradation,^[6]^ while mono‐ubiquitylation of p53 has been associated with nuclear‐cytoplasmic shuttling,^[7]^ mitochondrial translocation,^[8]^ and chromatin association.^[9]^ At first glance, it may seem surprising that mono‐ubiquitylation has different effects on p53. However, p53 can be ubiquitylated at different lysine residues and, thus, the eventual outcome likely depends on at which lysine p53 is mono‐ubiquitylated.

A prerequisite to prove the general hypothesis that the effect of mono‐ubiquitylation on the biochemical/biological properties of p53 depends on the actual site of modification is the availability of homogeneous populations of site‐specifically ubiquitylated p53.^[9]^ The generation of defined Ub‐protein conjugates by enzymatic means is rather challenging, since most, if not all, E3 ligases are promiscuous insofar as they frequently ubiquitylate substrate proteins at several lysine residues and mediate both mono‐ and poly‐ubiquitylation. Therefore, a number of chemical biological approaches to generate defined Ub‐protein conjugates have been developed, such as native chemical ligation,^[10]^ thiol‐based ligation,[^10b^, ^11^] Cu(I)‐catalyzed alkyne‐azide cycloaddition (CuAAC),^[12]^ oxime ligation,^[13]^ and sortylation.^[14]^ However, none of these methods has been applied to p53 so far.

We previously reported on the generation of defined Ub conjugates including Ub dimers and site‐specifically mono‐ubiquitylated forms of PCNA and the linker histone H1.2 by combining CuAAC with the genetic code expansion technology to incorporate unnatural amino acids at distinct positions of a protein of interest.[^12a^, ^12f^, ^12i^] To test whether this approach can also be applied to p53, we used the amber codon suppression (ACS) method to generate a p53 variant containing the pyrrolysine analog Plk at a distinct position and conjugated it via CuAAC to a Ub variant harboring azidohomoalanine at its C terminus. This resulted in reasonable amounts of p53‐Ub conjugates (Figure S1 in the Supporting Information). We then subjected p53‐Ub to ubiquitylation assays in the presence of HMD2 or the E3 ligase E6AP in complex with E6 oncoprotein of human papillomavirus type 16 to determine whether ubiquitylation at the desired position alters the property of p53 to serve as substrate for the two E3 ligases.^[15]^ However, respective control reactions revealed that the reaction conditions of CuAAC, i. e. the presence of copper, drive p53 into a conformation that is neither recognized by HDM2 nor by the E6‐E6AP complex (note that the structural requirements of p53 to serve as substrate for HDM2 and for the E6‐E6AP complex are clearly different)^[16]^ (Figure S1).

p53 is a zinc‐binding protein, and since copper does not interfere with ubiquitylation reactions per se (Figure S1C), we assumed that copper somehow affects the structural integrity of p53 as previously shown for other zinc‐binding proteins.^[17]^ We therefore switched to oxime ligation, a bioorthogonal conjugation strategy that does not require metal ions and can be performed under non‐denaturing conditions.^[18]^ In this reaction, an aminooxy group reacts with a carbonyl group to form an oxime linkage (Figure 1A). Furthermore, oxime ligation was used before to produce linkage‐specific Ub conjugates,^[13]^ as the oxime linkage is a reasonable mimic of the natural isopeptide bond (Figure S2). To equip p53 and Ub with the required functional groups at the desired positions, we used ACS again.^[19]^ In brief (for details, see Supporting Information), the lysine codon at position 120 of p53 was replaced by the amber stop codon enabling the introduction of a carbonyl group at this position via site‐specific incorporation of ketolysine (KeK).^[20]^ We chose this position, as K120 contributes to the sequence‐specific DNA binding capacity of p53^[21]^ and is known to be ubiquitylated,^[15]^ but whether K120 ubiquitylation effects DNA binding or other properties of p53 is unknown. *E. coli* cells harboring the respective expression plasmid and the required Pyrrolysine(Pyl)‐tRNA‐synthetase/tRNAPyl pair were cultured in presence of 10 mM KeK^[22]^ resulting in the expression of p53‐120KeK (Figure S3). Upon purification, the identity of p53‐120Kek was verified by LC‐MS/MS (Figure S6A).

**Figure 1:**
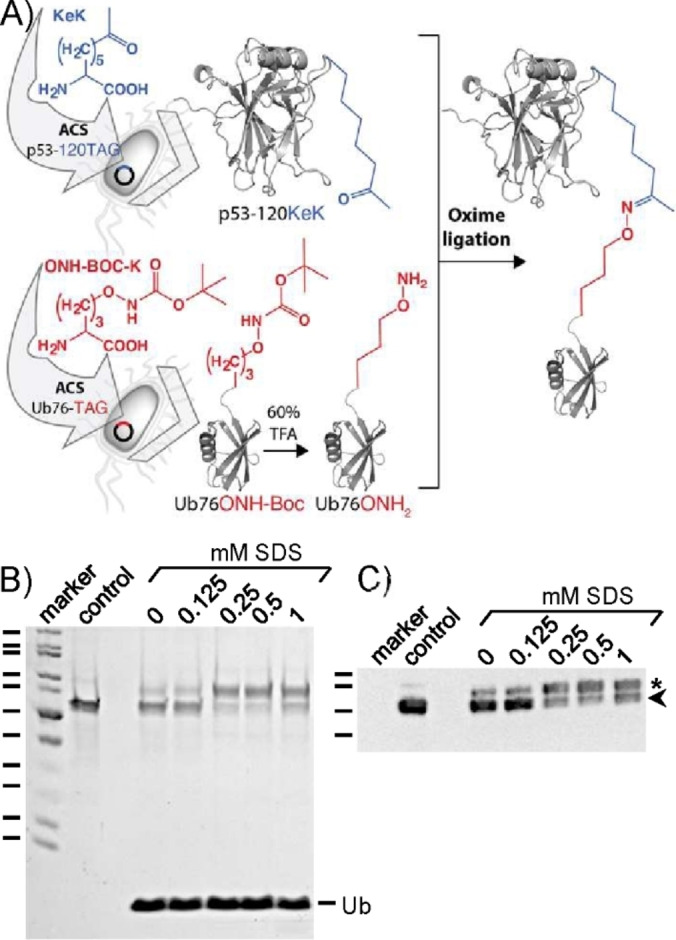
Generation of mono‐ubiquitylated p53 via oxime ligation. A) Schematic of the approach. For details, see text. B), C) Oxime ligation is enhanced with increasing amounts of non‐denaturing SDS concentrations. p53‐120KeK (20 pmol) was mixed with a 50‐molar excess of Ub76ONH_2_ in presence of increasing concentrations of SDS as indicated and subjected to two freeze‐thaw cycles^[18a]^ or, alternatively, to incubation for 20 h at 25 °C (Figure S4E). Reaction products were subjected to SDS‐PAGE followed by Coomassie blue staining (B) or by Western blot analysis using an anti‐p53 antibody (C). Control, reaction in the absence of Ub76ONH_2._ Running positions of p53‐120KeK and p53‐120‐Ub are indicated by an arrow and an asterisk, respectively. Running positions of molecular mass markers (kDa) are indicated from top to bottom in B): 120,100, 85, 70, 60, 50, 40, 30, 25, 20, 15, and in C): 70,60,50,40.

To equip Ub with a C‐terminal aminooxy group (Ub76ONH_2_) by ACS, the C‐terminal glycine codon at position 76 was replaced by the amber stop codon. As unnatural amino acid, we chose the lysine derivative Nϵ‐aminooxy‐L‐lysine, the aminooxy group of which had to be protected by an Nϵ‐latent protection group to prevent unwanted reactions with aldehyde or keto groups of endogenous bacterial molecules.^[13a]^ As Nϵ‐latent protection group, we chose the acid‐labile *tert*‐butoxycarbonyl (Boc) group that also served as recognition motif for the respective Pyl‐tRNA‐synthetase/tRNAPyl pair for incorporation of Nϵ‐aminooxy‐(Boc)‐L‐lysine (U1).^[13a]^ Upon expression in *E. coli* and subsequent purification (Figure S4), Ub76ONH‐Boc was deprotected by using 60 % TFA, and the identity of Ub76ONH_2_ was verified by LC‐MS (Figure S4C). This also showed that incorporation of U1 did not proceed to completion (Figure S4C), resulting in a mixture of truncated Ub (Ub75) and full‐length Ub (Ub76ONH‐Boc) (Figure S4C). Although the truncated form cannot be separated from full‐length Ub, this is not a concern since it cannot react with p53‐120KeK. Thus, upon oxime ligation, Ub75 can easily be removed from the p53‐Ub conjugate by anion exchange chromatography (for details, see Supporting Information).

To ensure that the functionality of p53 in the context of the envisioned p53‐Ub conjugate is affected solely by the presence of Ub and not by other factors, the oxime ligation reaction should be performed under near‐physiological conditions. However, oxime reactions proceed rather slowly at neutral pH.^[10]^ Since we previously showed that anionic surfactants enhance CuAAC bioconjugation reactions,^[12f]^ we tested whether addition of non‐denaturing concentrations of SDS also enhances oxime ligation. Indeed, increasing amounts of SDS resulted in increased yields of p53‐120‐Ub (Figure 1; Figure S4D, E) of up to 70 % of the p53 input. That the ligation product indeed represents p53‐120‐Ub was verified by LC‐MS/MS analysis (Figure S6B).

Upon further purification (Figure S5), we investigated the structural integrity of p53‐120‐Ub. Due to the fact that E6 recognizes p53 only when it is in a wild‐type (wt)‐like conformation,^[23]^ we determined whether the incorporation of KeK or the covalent attachment of Ub to position 120 affects the ability of p53 to serve as a substrate in in vitro ubiquitylation assays using the E6‐E6AP complex as E3 ubiquitin ligase (Figure 2A). Independent of the modification state, the p53 variants were recognized and ubiquitylated by the E6‐E6AP complex to a similar extent. Similarly, like wt p53, p53‐120KeK and p53‐120‐Ub were also ubiquitylated by HDM2 (Figure 2B). These data demonstrate that in contrast to CuAAC, the reaction conditions of oxime ligation do not affect the structural integrity of p53 and that mono‐ubiquitylation at position 120 does not affect the property of p53 to be recognized by the E3 ligases tested.

**Figure 2:**
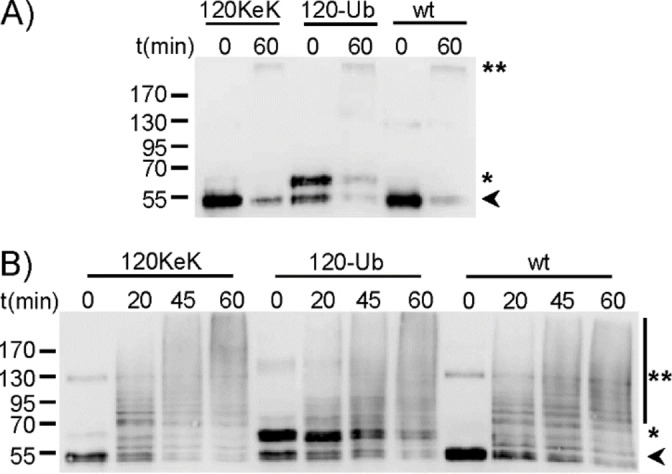
Ubiquitylation assays with p53 variants. Equal amounts of p53‐120‐Ub, p53‐120KeK, and wild‐type (wt) p53 were incubated with A) E6‐E6AP or B) HDM2 under in vitro ubiquitylation conditions for the times indicated. Reaction products were analyzed by SDS‐PAGE followed by Western blot analysis with an antibody directed against p53. Running positions of the non‐modified form of wt p53 and p53‐120Kek, p53‐120‐Ub, and (poly‐)ubiquitylated forms are indicated by an arrow, asterisk, and double asterisk, respectively. Running positions of molecular mass standards (kDa) are indicated on the left.

Acetylation of p53 at K120 was reported to affect the sequence‐specific DNA binding properties of p53.^[21]^ Therefore, we assessed the ability of p53‐120KeK and p53‐120‐Ub to bind to the p21 response element (p21‐RE) in electromobility shift assays (EMSA) in comparison to wt p53. Yet, no significant difference in the binding ability of the different p53 variants was observed (Figure 3A, Figure S7). While this further supports the notion that the reaction conditions of oxime ligation and the incorporation of KeK do not detectably affect the structural integrity of p53, it may seem surprising that attachment of a rather large molecule such as Ub to K120 does not interfere with DNA binding. However, it was recently reported that upon binding of p53 to so‐called high affinity RE such as p21‐RE, the side chain of K120 is disordered and not involved in hydrogen bonding with nucleobases.^[24]^ This provides a reasonable explanation for our observation that p53‐120‐Ub can still bind to the p21‐RE and indicates that p53‐120‐Ub adopts a wt p53‐like conformation.

**Figure 3:**
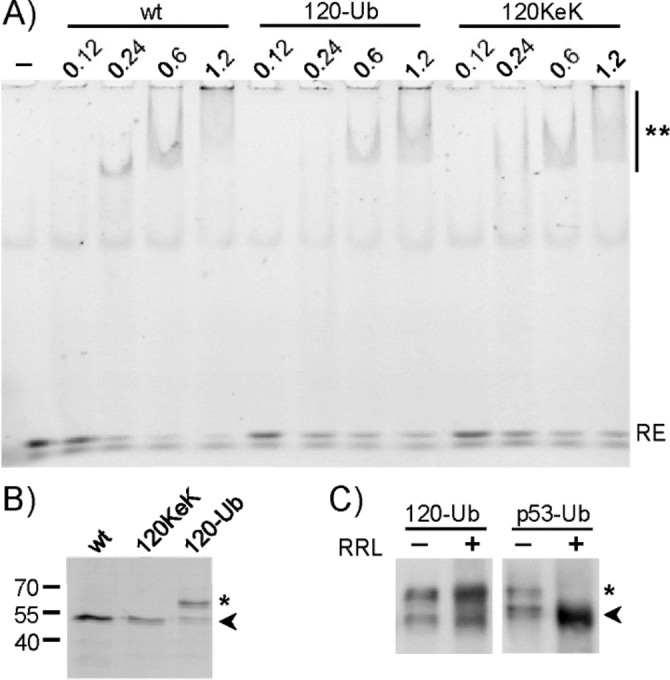
A) Characterization of the DNA binding ability of p53 variants by EMSA. Increasing concentrations of p53‐120‐Ub, p53‐120KeK, and wild‐type (wt) p53 were incubated with 10 nM fluorescein‐labeled p21 response element (RE) and analyzed on a 4 % native TBE polyacrylamide gel followed by fluorescence read out at 473 nm. ‐, control reaction in the absence of p53; RE, running position of the unbound RE; **, running position of the RE‐p53 complex. B) Loading control for the proteins used in A). Running positions of non‐modified p53/p53‐120KeK and p53‐120‐Ub are indicated by an arrow and an asterisk, respectively. Running positions of molecular mass standards (kDa) are indicated on the left. C) The oxime‐ linkage of p53‐120‐Ub is not hydrolyzed. 150 ng oxime‐linked p53‐120‐Ub or enzymatically mono‐ubiquitylated p53 were incubated in the absence or presence of 5 μL rabbit reticulocyte lysate (RRL) for 30 min at 37 °C. RRL served as a source of de‐ubiquitylating enzymes. Reaction products were analyzed by SDS‐PAGE followed by Western blot analysis with a p53‐specific antibody. The running position of p53‐120‐Ub/enzymatically mono‐ubiquitylated p53 and the non‐modified form of p53 is denoted with an asterisk and an arrow, respectively.

Mono‐ubiquitylation affects the intracellular localization of p53,[^7^, ^8^] which is likely due to changes in the protein‐protein interaction properties of mono‐ubiquitylated p53. Since cells or cell lysates are rich in de‐ubiquitylating enzymes (DUBs), Ub conjugates that are resistant to the action of DUBs would be a prerequisite for such interactome analyses. To test the stability of oxime‐linked p53‐120‐Ub, we incubated p53‐120‐Ub with rabbit reticulocyte lysate for 30 min at 37 °C (Figure 3C). As previously shown for oxime‐linked Ub‐Ub conjugates^[13a]^ and in contrast to enzymatically ubiquitylated p53, which served as control, the oxime‐linked p53‐120‐Ub was not hydrolyzed. Finally, we showed that the oxime ligation strategy is not limited to the generation of mono‐ubiquitylated forms of p53 but can also be applied to other proteins such as the linker histone H1.2 (Figure S8).

In conclusion, the combination of the genetic code expansion technology with oxime ligation enables the generation of mono‐ubiquitylated forms of p53 and other proteins in a site‐specific manner. Importantly, the reaction conditions of oxime ligation do not interfere per se with the structural integrity of p53 allowing to analyze known properties of p53 that may be affected by mono‐ubiquitylation. Furthermore, oxime‐linked p53‐Ub conjugates are resistant to the action of DUBs. Thus, they are well suited for affinity enrichment experiments to identify proteins that interact selectively with mono‐ubiquitylated forms of p53 thereby modulating the growth‐suppressive properties of p53.

## Conflict of interest

The authors declare no conflict of interest.

## Supporting information

As a service to our authors and readers, this journal provides supporting information supplied by the authors. Such materials are peer reviewed and may be re‐organized for online delivery, but are not copy‐edited or typeset. Technical support issues arising from supporting information (other than missing files) should be addressed to the authors.

Supporting Information

## Data Availability

The data that support the findings of this study are available in the supplementary material of this article.

## References

[1] V. J. Bykov , N. Issaeva , G. Selivanova , K. G. Wiman , Carcinogenesis 2002, 23, 2011–2018. 12507923 10.1093/carcin/23.12.2011

[2] D. P. Xirodimas , M. Scheffner , Subcell. Biochem. 2010, 54, 116–135. 21222278 10.1007/978-1-4419-6676-6_10

[3] O. Laptenko , C. Prives , Cell Death Differ. 2006, 13, 951–961. 16575405 10.1038/sj.cdd.4401916

[4] Y. Liu , O. Tavana , W. Gu , J. Mol. Cell Biol. 2019, 11, 564–577. 31282934 10.1093/jmcb/mjz060 PMC6736412

[5a] A. Hershko , A. Ciechanover , Annu. Rev. Biochem. 1998, 67, 425–479; 9759494 10.1146/annurev.biochem.67.1.425

[5b] D. Komander , M. Rape , Annu. Rev. Biochem. 2012, 81, 203–229. 22524316 10.1146/annurev-biochem-060310-170328

[6] M. S. Rodriguez , J. M. Desterro , S. Lain , D. P. Lane , R. T. Hay , Mol. Cell. Biol. 2000, 20, 8458–8467. 11046142 10.1128/mcb.20.22.8458-8467.2000 PMC102152

[7a] M. A. Lohrum , D. B. Woods , R. L. Ludwig , E. Balint , K. H. Vousden , Mol. Cell. Biol. 2001, 21, 8521–8532; 11713287 10.1128/MCB.21.24.8521-8532.2001 PMC100015

[7b] L. Nie , M. Sasaki , C. G. Maki , J. Biol. Chem. 2007, 282, 14616–14625. 17371868 10.1074/jbc.M610515200

[8] N. D. Marchenko , S. Wolff , S. Erster , K. Becker , U. M. Moll , EMBO J. 2007, 26, 923–934. 17268548 10.1038/sj.emboj.7601560 PMC1852828

[9] L. Le Cam , L. K. Linares , C. Paul , E. Julien , M. Lacroix , E. Hatchi , R. Triboulet , G. Bossis , A. Shmueli , M. S. Rodriguez , O. Coux , C. Sardet , Cell 2006, 127, 775–788. 17110336 10.1016/j.cell.2006.09.031

[10a] C. P. Hackenberger , D. Schwarzer , Angew. Chem. Int. Ed. 2008, 47, 10030–10074; 10.1002/anie.200801313 19072788

[10b] P. E. Dawson , T. W. Muir , I. Clark-Lewis , S. B. Kent , Science 1994, 266, 776–779. 7973629 10.1126/science.7973629

[11a] L. Yin , B. Krantz , N. S. Russell , S. Deshpande , K. D. Wilkinson , Biochemistry 2000, 39, 10001–10010; 10933821 10.1021/bi0007019

[11b] R. E. Morgan , V. Chudasama , P. Moody , M. E. Smith , S. Caddick , Org. Biomol. Chem. 2015, 13, 4165–4168; 25736233 10.1039/c5ob00130g PMC4372856

[11c] F. Meier , T. Abeywardana , A. Dhall , N. P. Marotta , J. Varkey , R. Langen , C. Chatterjee , M. R. Pratt , J. Am. Chem. Soc. 2012, 134, 5468–5471; 22404520 10.1021/ja300094r PMC3315850

[11d] L. Long , M. Furgason , T. Yao , Methods 2014, 70, 134–138; 25063569 10.1016/j.ymeth.2014.07.006 PMC4268123

[11e] C. Chatterjee , R. K. McGinty , B. Fierz , T. W. Muir , Nat. Chem. Biol. 2010, 6, 267–269; 20208522 10.1038/nchembio.315

[11f] T. Abeywardana , Y. H. Lin , R. Rott , S. Engelender , M. R. Pratt , Chem. Biol. 2013, 20, 1207–1213. 24210006 10.1016/j.chembiol.2013.09.009 PMC3855323

[12a] S. Eger , B. Castrec , U. Hübscher , M. Scheffner , M. Rubini , A. Marx , ChemBioChem 2011, 12, 2807–2812; 22052741 10.1002/cbic.201100444

[12b] S. Eger , M. Scheffner , A. Marx , M. Rubini , Methods Mol. Biol. 2012, 832, 589–596; 22350914 10.1007/978-1-61779-474-2_41

[12c] N. D. Weikart , S. Sommer , H. D. Mootz , Chem. Commun. 2012, 48, 296–298; 10.1039/c1cc15834a 22095407

[12d] D. Schneider , T. Schneider , D. Rösner , M. Scheffner , A. Marx , Bioorg. Med. Chem. 2013, 21, 3430–3435; 23611767 10.1016/j.bmc.2013.03.063

[12e] D. Rösner , T. Schneider , D. Schneider , M. Scheffner , A. Marx , Nat. Protoc. 2015, 10, 1594–1611; 26401915 10.1038/nprot.2015.106

[12f] D. Schneider , T. Schneider , J. Aschenbrenner , F. Mortensen , M. Scheffner , A. Marx , Bioorg. Med. Chem. 2016, 24, 995–1001; 26827138 10.1016/j.bmc.2016.01.027

[12g] X. Zhao , J. Lutz , E. Höllmüller , M. Scheffner , A. Marx , F. Stengel , Angew. Chem. Int. Ed. 2017, 56, 15764–15768; 10.1002/anie.201705898 29045006

[12h] V. Hagmann , S. Sommer , P. Fabian , J. Bierlmeier , N. van Treel , H. D. Mootz , D. Schwarzer , J. E. Azevedo , G. Dodt , Sci. Rep. 2018, 8, 16014; 30375424 10.1038/s41598-018-34200-5 PMC6207756

[12i] E. Höllmüller , S. Geigges , M. L. Niedermeier , K. M. Kammer , S. M. Kienle , D. Rosner , M. Scheffner , A. Marx , F. Stengel , Nat. Commun. 2021, 12, 3497. 34108453 10.1038/s41467-021-23636-5 PMC8190259

[13a] M. Stanley , S. Virdee , ChemBioChem 2016, 17, 1472–1480; 27197715 10.1002/cbic.201600138 PMC5094518

[13b] A. Shanmugham , A. Fish , M. P. Luna-Vargas , A. C. Faesen , F. El Oualid , T. K. Sixma , H. Ovaa , J. Am. Chem. Soc. 2010, 132, 8834–8835; 20540574 10.1021/ja101803s

[13c] S. K. Singh , I. Sahu , S. M. Mali , H. P. Hemantha , O. Kleifeld , M. H. Glickman , A. Brik , J. Am. Chem. Soc. 2016, 138, 16004–16015. 27960333 10.1021/jacs.6b09611

[14] M. Fottner , A. D. Brunner , V. Bittl , D. Horn-Ghetko , A. Jussupow , V. R. I. Kaila , A. Bremm , K. Lang , Nat. Chem. Biol. 2019, 15, 276–284. 30770915 10.1038/s41589-019-0227-4

[15] W. M. Chan , M. C. Mak , T. K. Fung , A. Lau , W. Y. Siu , R. Y. Poon , Mol. Cancer Res. 2006, 4, 15–25. 16446403 10.1158/1541-7786.MCR-05-0097

[16] E. A. Medcalf , J. Milner , Oncogene 1993, 8, 2847–2851. 7690928

[17] Y. Kim , S. H. Kim , D. Ferracane , J. A. Katzenellenbogen , C. M. Schroeder , Bioconjugate Chem. 2012, 23, 1891–1901. 10.1021/bc300262h PMC3462365 22871171

[18a] S. M. Agten , D. P. Suylen , T. M. Hackeng , Bioconjugate Chem. 2016, 27, 42–46; 10.1021/acs.bioconjchem.5b00611 26649643

[18b] S. M. Agten , P. E. Dawson , T. M. Hackeng , J. Pept. Sci. 2016, 22, 271–279; 27006095 10.1002/psc.2874

[18c] D. K. Kolmel , E. T. Kool , Chem. Rev. 2017, 117, 10358–10376; 28640998 10.1021/acs.chemrev.7b00090 PMC5580355

[18d] S. Wang , G. N. Nawale , S. Kadekar , O. P. Oommen , N. K. Jena , S. Chakraborty , J. Hilborn , O. P. Varghese , Sci. Rep. 2018, 8, 2193. 29391582 10.1038/s41598-018-20735-0 PMC5794741

[19] J. W. Chin , Annu. Rev. Biochem. 2014, 83, 379–408. 24555827 10.1146/annurev-biochem-060713-035737

[20] Y. Huang , W. Wan , W. K. Russell , P. J. Pai , Z. Wang , D. H. Russell , W. Liu , Bioorg. Med. Chem. Lett. 2010, 20, 878–880. 20074948 10.1016/j.bmcl.2009.12.077

[21a] R. Vainer , S. Cohen , A. Shahar , R. Zarivach , E. Arbely , J. Mol. Biol. 2016, 428, 3013–3025; 27338200 10.1016/j.jmb.2016.06.009

[21b] E. Arbely , E. Natan , T. Brandt , M. D. Allen , D. B. Veprintsev , C. V. Robinson , J. W. Chin , A. C. Joerger , A. R. Fersht , Proc. Natl. Acad. Sci. USA 2011, 108, 8251–8256. 21525412 10.1073/pnas.1105028108 PMC3100949

[22] H. Neumann , S. Y. Peak-Chew , J. W. Chin , Nat. Chem. Biol. 2008, 4, 232–234. 18278036 10.1038/nchembio.73

[23] M. Scheffner , T. Takahashi , J. M. Huibregtse , J. D. Minna , P. M. Howley , J. Virol. 1992, 66, 5100–5105. 1321290 10.1128/jvi.66.8.5100-5105.1992 PMC241378

[24] M. Farkas , H. Hashimoto , Y. Bi , R. V. Davuluri , L. Resnick-Silverman , J. J. Manfredi , E. W. Debler , S. B. McMahon , Nat. Commun. 2021, 12, 484. 33473123 10.1038/s41467-020-20783-z PMC7817693

