# Supplementary material for: Generation and Characterization of Site‐Specifically Mono‐Ubiquitylated p53
Source: Chembiochem. 2022 Feb 3;23(6):e202100659. doi: 10.1002/cbic.202100659 (PMC9303418; doi:10.1002/cbic.202100659)
Supplement: Supplementary file 1 — Supporting Information [file CBIC-23-0-s001.pdf]

ChemBioChem

Supporting Information

Generation and Characterization of Site-Specifically Mono-Ubiquitylated p53

Alexandra Julier, Vanessa Radtke, Andreas Marx,* and Martin Scheffner*

1. General Information

Chemical reagents were obtained from ABCR, Merck/Sigma Aldrich, Roth, Bachem, TCI, Acros Organics, Fluka, Riedel de Haen, Iris Bioctech GmbH or VWR. Milli-Q (MQ) H_2O was obtained by the Milli-Q system (Millipore Corporation).

MPLC was performed on a PrepChrom C-700 system (Büchi) or SepacoreR Flash System X (Büchi). RP-columns were prepacked (25-40 microns; Götec).

NMR spectra were recorded at ambient temperature with Bruker Avance III 400 instrument. Samples were prepared in deuterated solvents from Deutero or Eurisotop. The chemical shift δ are reported in parts per million (ppm) using 1H or 13C solvent signal as reference. Multiplicity was assigned as singlet (s), doublet (d), triplet (t), quadruplet (q), pentuplet (p), or multiplet (m).

LC-MS high-resolution mass spectra (HR-MS) were obtained on a Bruker Daltonics microTOF II instrument. LC-MS/MS spectra were recorded by the Proteomics Center, University of Konstanz.

2. Synthesis Protocols

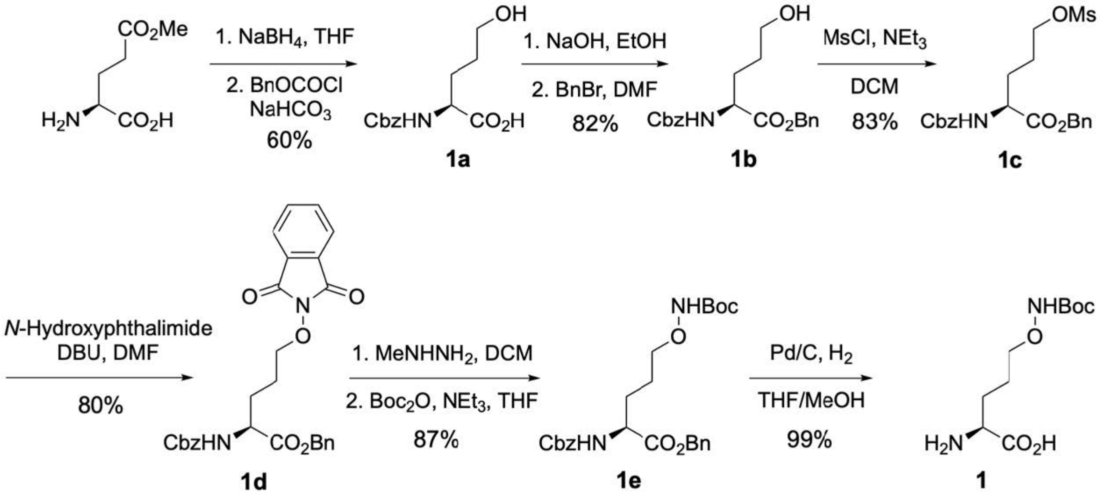

The synthesis of Nε-aminooxy-(tert-butoxycarbonyl)-L-lysine derivative **1** (termed **U1**) was accomplished in a 6-step route. The synthesis of compounds **1a**–**e** was published previously.[1] Different to the published protocols, the attachment of the Cbz protection group to prepare **1a** was performed in saturated $NaHCO_3$, since the use of 0.2 M $NaHCO_3$ did not result in product formation. The removal of the benzyl ester and the Cbz group were performed with 20 mol% Pd/C by hydrogenolysis with a yield of 99 %.

^{1}H NMR (400 MHz, Deuterium Oxide) δ = 3.95 (t, *J* = 6.1 Hz, 2H, δ-CH_2), 3.82 (t, *J* = 6.1 Hz, 1H, α-C**H**), 2.09–1.94 (m, 2H, β-CH_2), 1.87–1.70 (m, 2H, γ-CH_2), 1.53 (s, 9H, C(CH_3)$_3$) ppm.

^{13}C NMR (400 MHz, Deuterium Oxide) δ = 175.86 (**C**O_2H), 159.83 (NH**C**O), 84.89 (**C**(CH_3)$_3$), 77.29 (δ-**C**), 55.97 (α-**C**), 28.95 (C(**C**H_3)$_3$), 28.61 (β-**C**), 24.67 (γ-**C**) ppm.

HR-MS [M+H+] (m/z): observed: 249.1441, calculated: 249.1445, deviation: 1.6 ppm.

The synthesis of KeK was performed as described.[2]

^{1}H NMR (400 MHz, Deuterium Oxide) δ = 4.06 (t, *J* = 6.2 Hz, 2H, a-C**H**), 2.62 (t, *J* = 7.3 Hz, 2H, ζ-CH_2), 2.25 (s, 3H, θ-CH_3), 2.02–1.93 (m, 2H, β-CH_2), 1.61 (p, 2H, *J* = 7.4 Hz, ε-CH_2),1.58–1.37 (m, 4H, γ-CH_2, δ-CH_2) ppm.

^{13}C NMR (400 MHz, Deuterium Oxide) δ = 217.44 (η-**C**O), 172.80 (**C**O_2H), 53.27 (a-**C**), 43.06 (ζ-**C**), 29.67 (β-**C**), 29.32 (θ-**C**), 27.70 (δ-**C**), 23.90 (γ-**C**), 22.87 (ε-**C**) ppm.

3. NMR Spectra

^{1}H NMR

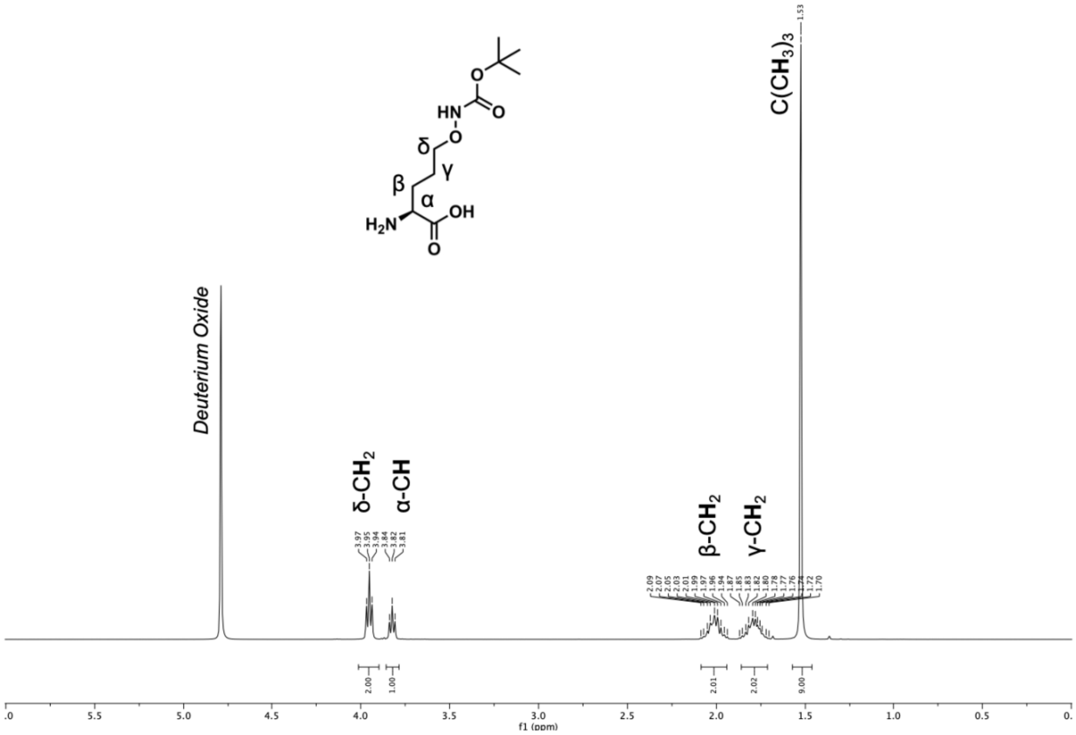

^{13}C NMR

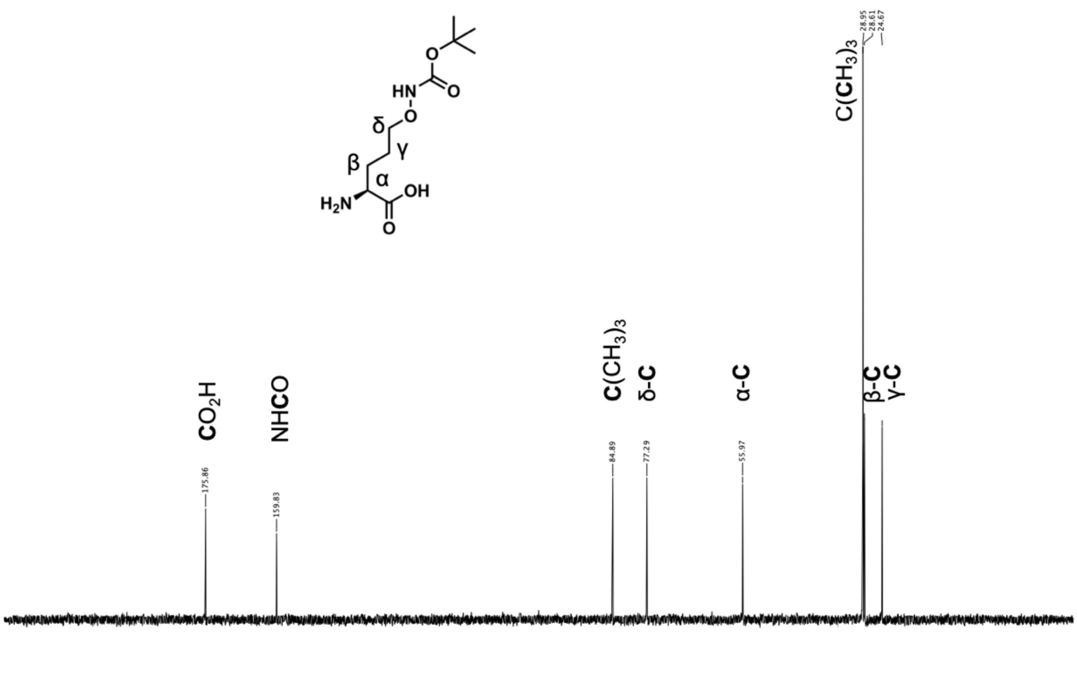

^{1}H NMR

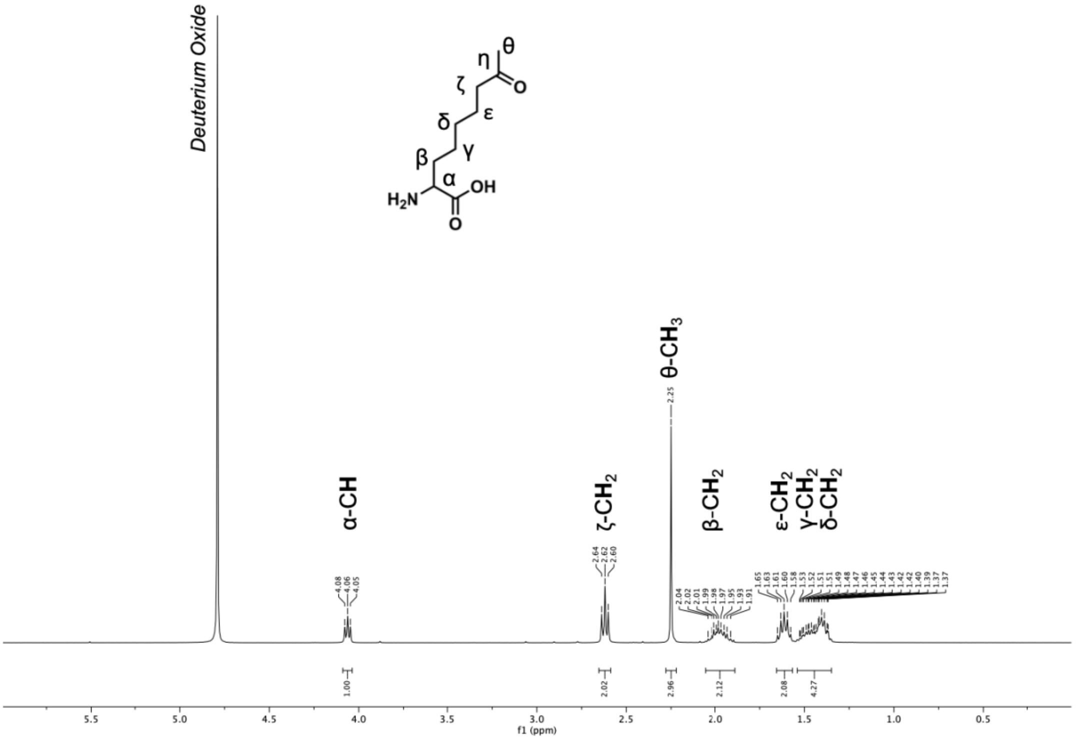

^{13}C NMR

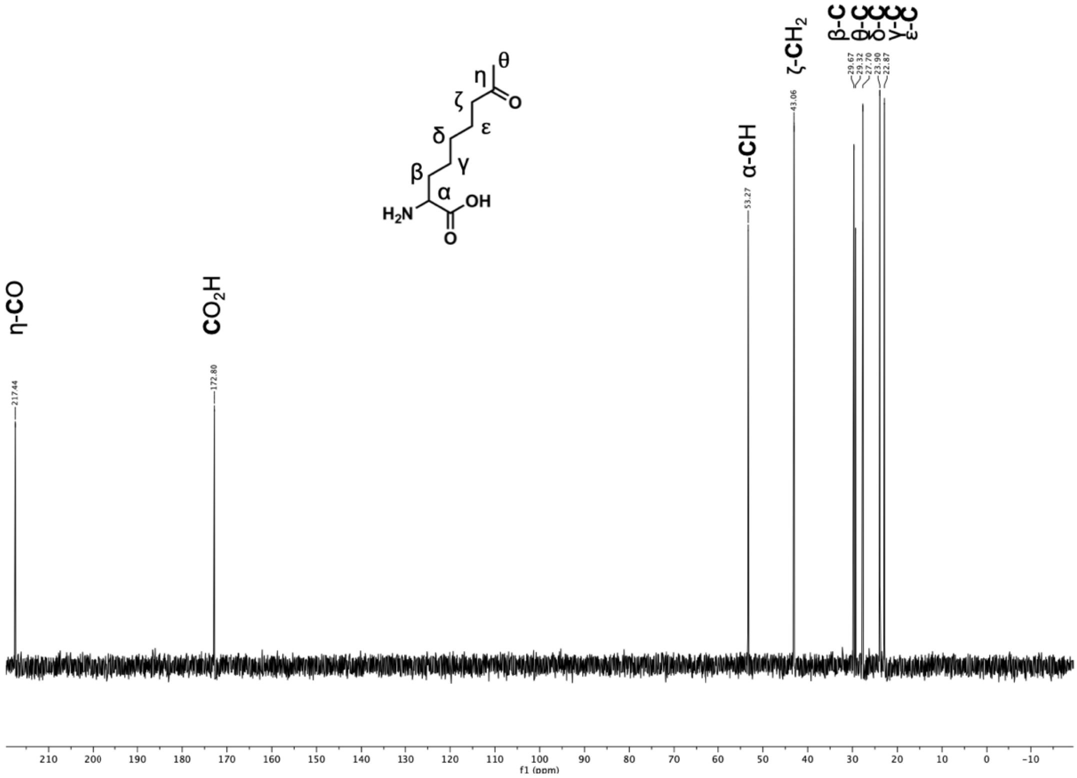

4. Experimental Section

Expression and purification of alkyne-functionalized p53 (p53-372Plk)

The cDNA encoding human p53 372TAG (replacement of lysine codon 372 by stop codon TAG) was cloned into pGEX-2TK, which additionally harbors the Pyl-tRNA cassette of *M. barkeri*,[3] and co-transformed with pRSF-duet encoding the Pyl-tRNA synthetase of *M. barkeri*[3] in *E. coli* BL21 DE3. For expression of the GST-p53 fusion protein, a single clone was inoculated in 30 mL LB medium supplemented with the appropriate antibiotics and cells were grown overnight. The pre-culture was diluted to OD_{600} = 0.1 in 1 L LB supplemented with the appropriate antibiotics. At OD_{600} = 0.3-0.4, 3 mM (final concentration) Plk were added and cells further cultivated to OD_{600}= 0.6-0.8. Gene expression was induced by adding 1 mM IPTG and cells were incubated at 25 °C overnight. After cell harvest, the pellet was resuspended in 30 mL 1x PBS containing 1 % Triton X-100, 1 mM DTT, 1 µg/mL aprotinin and leupeptin, 100 µM Pefabloc, and sonicated (Branson Sonifier 250; 4x 20 pulses, duty cycle 20 %, output control 3-4). Cell debris was removed by centrifugation (27,000x g, 4 °C, 20 min). The supernatant was transferred into a fresh tube and incubated at 4 °C for 90 min under continuous shaking with Glutathione Sepharose 4B (GE Healthcare; ratio beads : supernatant = 1:1000, v/v) equilibrated in 1x PBS, 1 % Triton X-100. After centrifugation at 500x g at 4 °C for 10 min, the supernatant was discarded. Beads were washed 3 times with 15 mL 1x PBS, 1 % Triton and once with 15 mL 25 mM Tris-HCl pH 7.5, 150 mM NaCl at 4 °C for 5 min. Then, beads were collected by centrifugation (500x g, 4 °C, 10 min) and the supernatant was discarded. p53 was eluted from the beads by addition of thrombin that cleaves between GST and p53 and incubation at 4 °C. The resulting tag-free p53 was loaded onto a 1 mL Heparin column and eluted with a gradient of 25 column volumes from 15 % buffer A (40 mM Tris-HCl pH 7.5, 0.01 % (v/v) NP-40, 2 mM DTT) to 100 % buffer B (40 mM Tris-HCl pH 7.5, 1 M NaCl, 0.01 % (v/v) NP-40, 2 mM DTT). Fractions containing p53 were pooled followed by dialysis against storage buffer (40 mM Tris-HCl pH 8.0, 50 mM NaCl, 0.01 % (v/v) NP-40, 2 mM DTT, 10 % glycerol), flash-frozen with liquid nitrogen, and stored at -80 °C.

Expression and purification of UbG75Aha

Monofunctionalized UbG75Aha was produced as described.[4]

Generation of p53-372Ub by CuAAC

p53-372Plk (1 eq) in storage buffer was mixed with UbG75Aha (3-10 eq) in 1x PBS in presence of 1 mM SDS. The mixture was flushed with argon. THPTA (2 mM) and Cu(I) (1 mM) were added followed by flushing with argon to prevent Cu(I)-induced protein oxidation. After incubation at 4 °C for 30 min, the reaction was stopped with 10 mM EDTA pH 8.0. Reaction products were dialyzed against 40 mM Tris-HCl pH 8.0, 150 mM NaCl, 1 mM DTT.

Expression and purification of Ub76ONH-Boc (Ub76U1)

The cDNA encoding human Ub G76TAG (replacement of glycine codon 76 by stop codon TAG) was cloned into pET11a, which additionally encodes the Pyl-tRNA cassette of *M. barkeri*,[3] and co-transformed with pRSF-duet Pyl-tRNA synthetase 349W of *M. barkeri*[5] in *E. coli* BL21 DE3. For expression, a single clone was inoculated in LB medium supplemented with the appropriate antibiotics and cells were grown overnight. The pre-culture was diluted in LB medium supplemented with the appropriate antibiotics to OD_{600} = 0.1 and cultivated to OD_{600} = 0.3-0.4. Then, U1 was added to a final concentration of 1 mM. At OD_{600} = 0.6, gene expression was induced by adding 1 mM IPTG. Cells were cultivated overnight at 25 °C. After cell harvest, the pellet was resuspended in 1x PBS containing 1 % Triton X-100, 1 mM DTT, 1 µg/mL aprotinin and leupeptin, 100 µM Pefabloc, and sonicated (Branson Sonifier 250; 4x 20 pulses, duty cycle 20%, output control 3-4). Cell debris was removed by centrifugation (27,000x g, 4 °C, 20 min). The supernatant was precleared using an anion exchange column at neutral pH, and the flow-through was loaded onto HiLoad Superdex 75 pg 26/600 (GE Healthcare) equilibrated with 1x PBS using the ÄKTA purifier FPLC system. Protein was eluted with 1x PBS using an isocratic gradient. Fractions were analyzed by Tricine-PAGE and Coomassie blue staining.

Deprotection of purified Ub76ONH-Boc

Ub76ONH-Boc was deprotected by addition of TFA (final conc. 60 % (v/v)) followed by incubation at 37 °C for 2 h. Subsequent ice-cold ether precipitation and lyophilization afforded unprotected Ub76ONH$_2$. Lyophilized Ub76ONH$_2$ was dissolved in 1x PBS, aliquoted, and stored at -20 °C.

Expression and purification of p53-120KeK

To test whether oxime ligation is a valid strategy for the generation of mono-ubiquitylated p53 variants, we switched to position 120 of p53. Besides the notion that K120 is known to be ubiquitylated and to contribute to the DNA binding capabilities of p53, we chose this position, because in most cases ACS is not quantitative resulting in a mixture of full-length and truncated versions of the protein of interest. The more N-terminal the truncation occurs, the easier is the separation of the full-length product from the truncated product, e.g. by size exclusion chromatography or, as in this case, by heparin affinity chromatography (as binding of p53 to heparin depends on the presence of its DNA binding domain which comprises approximately residues 100-300).

A codon-optimized cDNA encoding human full-length p53 was cloned into a pGEX backbone containing the Pyl-tRNA cassette and an N-terminal His$_9$-lipoyl domain (HLD) tag[6] instead of the GST tag. The lysine codon at position 120 was replaced by the amber codon TAG by site-directed mutagenesis. *E. coli* BL21 DE3 were co-transformed with pGEX-HLD p53-120TAG and pRSF-duet1 containing the AcK-RS tRNA mutant of *M. barkeri*.[7] A single clone was inoculated in LB medium supplemented with the appropriate antibiotics and cultivated to OD_{600} = 1. The pre-culture was diluted in LB medium supplemented with the appropriate antibiotics to OD_{600} = 0.1 and cultivated at 37 °C. At OD_{600} = 0.3, Ketolysine (KeK) was added to a final concentration of 10 mM, and at OD_{600} = 0.6-0.8 expression was induced by adding 1 mM IPTG. Cells were cultivated at 25 °C for 20 h; afterwards cells were harvested by centrifugation. Cells were lysed in 50 mM phosphate buffer pH 8.0, 300 mM NaCl, 0.01 % (v/v) NP-40, 1 µg/mL aprotinin and leupeptin, 100 µM Pefabloc, 10 mM DTT. The lysate was cleared by centrifugation (15,000x g, 4 °C, 20 min) and the supernatant was loaded onto a Ni-NTA column. HLD-tagged p53 was eluted with a gradient of 25 column volumes from His-Trap p53 buffer A (50 mM phosphate buffer pH 8.0, 150 mM NaCl, 0.01 % (v/v) NP-40, 2 mM DTT, 20 mM imidazole) to 100 % His-Trap p53 buffer B (50 mM phosphate buffer pH 8.0, 150 mM NaCl, 0.01 % (v/v) NP-40, 2 mM DTT, 1 M imidazole) and dialyzed overnight in p53 dialysis buffer (50 mM phosphate buffer pH 7.2, 150 mM NaCl, 0.01 % (v/v) NP-40, 2mM DTT) at 4 °C in the presence of thrombin (Sigma-Aldrich), as thrombin cleaves between the HLD tag and p53. p53 was further purified by affinity chromatography on a heparin column. p53 was eluted from the heparin column with a gradient over 25 column volumes from 15 % heparin p53 buffer A (50 mM phosphate buffer pH 7.2, 0.01 % (v/v) NP-40, 2 mM DTT) to 100% heparin p53 buffer B (50 mM phosphate buffer pH 7.2, 1 M NaCl, 0.01 % (v/v) NP-40, 2 mM DTT). Fractions containing purified p53 were pooled followed by dialysis against p53 storage buffer (50 mM phosphate buffer pH 7.2, 150 mM NaCl, 0.01 % (v/v) NP-40, 2 mM DTT, 10 % glycerol), flash-frozen with liquid nitrogen and stored at -80 °C.

Expression, purification and functionalization of Ub75C-Keto

The cDNA encoding Ub75C (replacement of glycine codon 75 by cysteine codon) was cloned into pET3a. Ub75C was expressed in *E. coli* BL21 DE3. A single clone was inoculated in LB medium supplemented with the appropriate antibiotics and cultivated to OD_{600} = 1. The pre-culture was diluted in LB supplemented with the appropriate antibiotics to OD_{600} = 0.1 and cultivated at 37 °C to OD_{600} = 0.5-0.8. Gene expression was induced with 1 mM IPTG, and upon incubation overnight at 25 °C cells were harvested by centrifugation. Cells were resuspended in 20 mM sodium acetate buffer pH 4.5, lysed by sonication, and centrifuged (27,000x g, 4 °C, 20 min). The extract was heated to 70 °C for 20 min. After centrifugation, the supernatant was purified using 1 mL HiTrap SP Sepharose High Performance column (GE Healthcare). Ub75C was eluted using a linear gradient of NaCl from 25 mM to 1000 mM in 25 mM NaOAc, pH 4.0, 1 mm DTT. Ub75C containing fractions were pooled and chromatographed on a HiLoad Superdex 75 pg 26/600 (GE Healthcare) equilibrated with 1x PBS using

the ÄKTA purifier FPLC system. Protein was eluted with 1x PBS using an isocratic gradient. Ub75C containing fractions were pooled and concentrated. A 100 µM solution of Ub75C in PBS pH 7.2 was treated with 1 mM TCEP for 30 min at 37 °C. Afterwards, reduced Ub75C was diluted 5-fold with 1x PBS pH 7.2 supplemented with 1000 eq. chloroacetone (Keto) and incubated by shaking for 90 min at 25 °C. To monitor the reaction, 20 µL of the reaction were incubated with 25 eq. fluorescein maleimide in the dark (10 min at room temperature) and analyzed by SDS-PAGE followed by measuring fluorescence intensity on an FLA Imager at 473 nm and by Coomassie blue staining. The reaction mixture was dialyzed against 25 % MeOH/1x PBS pH 7.2, then 2.5 % MeOH/1x PBS pH7.2 and finally against 0.1x PBS pH 7.2. The protein solution was concentrated and stored at -20°C. The molecular mass of Ub75C-Keto was determined by HR-MS.

Expression, purification, and deprotection of Histone 1.2-206U1

The cDNA encoding Histone 1.2 (H1.2)-206TAG (replacement of lysine codon 206 by stop codon TAG) harboring a C-terminal His_6-tag was cloned into pET11a, which additionally encodes the Pyl-tRNA cassette of *M. barkeri*, and co-transformed with pRSF-duet Pyl-tRNA-synthetase 349F of *M. barkeri* in *E. coli* BL21 DE3. At OD_{600} = 0.2-0.3, **U1** (final concentration 1 mM) was added and expression was induced by addition of IPTG (final concentration 1 mM) when OD_{600} = 0.6-0.9 was reached. After expression at 37 °C for 20 h, cells were pelleted and resuspended. After sonication, the mixture was centrifuged and washed several times. Inclusion bodies were solubilized by incubation with 50 mM Tris-HCl pH 7.0, 6 M urea, 1 M NaCl, 10 mM β-mercaptoethanol at 4 °C overnight. After centrifugation, the supernatant was purified by immobilized metal ion chromatography with cOmpleteTM His-Tag Purification Resin (Roche). Fractions containing H1.2-206U1 were identified by SDS-PAGE and Coomassie blue staining, pooled and dialyzed against MQ H_2O. The yield was 0.49 mg of H1.2-206U1 per L culture. For deprotection, H1.2-206U1 was mixed with TFA (67 % final concentration) and incubated for 2 h at 23 °C and continuous shaking (250 rpm). The protein was precipitated in diethyl ether and centrifuged (10,000 rpm, 20 min, 4 °C). The resulting H1.2-206ONH_2 was dissolved in MQ H_2O.

Oxime Ligation

Incorporation of U1 into ubiquitin did not proceed to completion (Figure S4 C), resulting in a mixture of truncated Ub (Ub75) and full-length Ub76ONH-Boc. Thus, for oxime ligation, a mixture consisting of Ub75 and Ub76ONH_2 (1 nmol) was mixed with p53-120KeK (20 pmol) in p53 buffer (50 mM phosphate buffer pH 7.2, 150 mM NaCl, 0.01 % (v/v) NP-40, 2 mM DTT) containing 0.5 mM SDS followed by incubation at 25 °C for 20 h (Fig. S4 E) or by 2 freeze-thaw cycles with -20 °C as freezing temperature (Fig. 1). The reaction products were analyzed by SDS-PAGE followed by Coomassie brilliant blue staining or Western blotting against p53 using the anti-p53 antibody DO-1. While the efficiency of oxime ligation was similar for both procedures (incubation at 25 °C, freeze-thaw cycles), the freeze-thaw cycle protocol was generally used for reasons of time and to exclude the possibility that the extended incubation time at 25 °C does not negatively affect p53 properties.

For generation of oxime-linked H1.2-206Ub, 50 µM Ub75C-Keto were mixed with 10 µM H1.2-206U1 and 2 mM SDS at pH 7.0 followed by 3 freeze-thaw cycles. The reaction mixture was analyzed by SDS-PAGE followed by Coomassie brilliant blue staining.

Purification of oxime-linked p53-120-Ub

For purification of p53-120-Ub, the reaction mixture was added to a heparin column (200 µl, Repligen) equilibrated in buffer A (50 mM phosphate buffer pH 7.2, 150 mM NaCl, 0.01 % (v/v) NP-40, 2 mM DTT). The flow-through of the heparin column was loaded onto a Q Sepharose column (50 µL). Upon loading, the column was washed with 5 column volumes of buffer A. Protein was eluted in a step gradient from 200-1000 mM NaCl. Fractions were examined by SDS-PAGE followed by Coomassie brilliant blue staining. As p53 mainly exists in a tetrameric form, the advantage of this procedure is that tetramers consisting mainly of p53-120-Ub subunits can be separated from tetramers mainly

consisting of non-modified p53-120KeK, as the latter almost quantitatively bind to heparin (see Fig. S5).

In vitro ubiquitylation

For in vitro ubiquitylation, 20-50 ng of recombinant p53 variants, 150 ng of baculovirus-expressed E1, 150 ng of UbcH5b, 5 µg of ubiquitin were incubated in the presence or absence of 200 ng Hdm2 or 200 ng of bacterially expressed E6AP and 200 ng GST- E6 in 25 mM Tris-HCl pH 7.5, 1 mM DTT, 2 mM ATP, 4 mM $MgCl_2$ in a total reaction volume of 30 µL. After incubation at 30 °C for 0 to 90 min, total reaction mixtures were stopped by addition of 5x Laemmli buffer and boiling at 95 °C for 5 min. Samples were analyzed by SDS-PAGE followed by Western blot analysis using an anti-p53 antibody (DO-1).
Expression and purification of E1 (UBA1), UbcH5b, Hdm2, E6AP, and a GST fusion protein of the E6 protein of human papillomavirus type 16 were performed as described.[8]

EMSA

For electrophoretic mobility shift assay (EMSA), a 4 % (v/v) native gel was casted using 10x TBE buffer (0.5 M Tris, 0.5 M Boric acid, 10 mM EDTA) and pre-equilibrated by running for 30 min at 200 V (4 °C) in TBE running buffer (25 mM Tris, 25 mM Boric acid, 0.5 mM EDTA) supplemented with 0.02 % IGEPAL. 0.1 % orange G in 10 % glycerol was used as loading dye. A dilution series of recombinant p53 (0.06, 0.12, 0.24, 0.6 and 1.2 pmol) was incubated with 400 fmol of fluorescein-labeled DNA representing the p53 response element of the p21 gene at 25 °C for 30 min. Subsequently, samples were loaded onto the native gel and the gel was run at 200 V for 90 min at 4 °C. Detection of the fluorescein-labeled DNA was performed on FUJIFILM FLA 5000, LBP channel using 473 nm excitation.

5. Supporting Figures

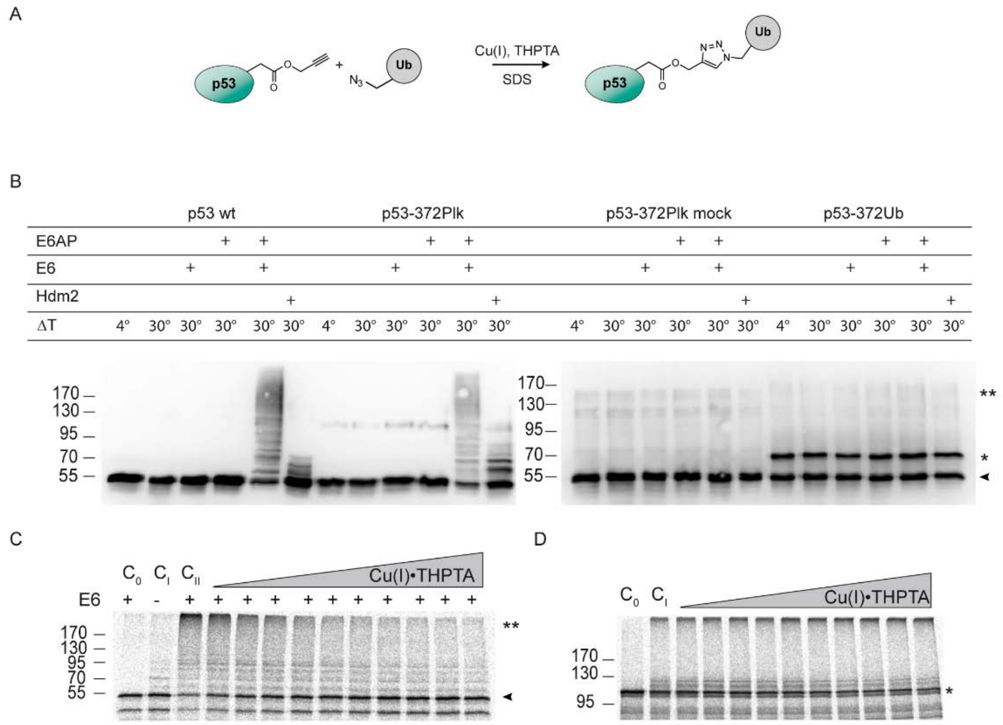

Figure S1. A) Schematic of the generation of ubiquitin (Ub)-modified p53 by CuAAC. B) The p53 variants indicated were incubated with E6-E6AP or Hdm2 under ubiquitylation conditions. Reaction products were analyzed by SDS-PAGE followed by Western blot analysis with the anti-p53 antibody DO-1. Running positions of non-modified p53 (arrow), mono-ubiquitylated p53 (asterisk), poly-ubiquitylated p53 (double asterisk), and molecular mass standards (kDa) are indicated. p53 wt, non-modified p53; p53-372Plk, p53 variant containing the unnatural amino acid Plk at position 372; p53-372Plk mock, p53-372Plk treated under CuAAC conditions in the absence of UbG75Aha; p53-372Ub, p53 modified at position 372 with UbG75Aha by CuAAC. C) Influence of Cu(I) on the ubiquitylation of in vitro translated p53 by E6-E6AP. In vitro translated, radiolabeled p53 (p53 wt) was incubated for 90 min with E6AP in the absence (C_I) or presence (C_{II}) of E6 under standard ubiquitylation conditions with increasing concentrations of Cu(I)•THPTA (4 µM-40 µM) as indicated. C_0, reaction for 90 min at 4 °C in the absence of E6AP. Reaction products were analyzed by SDS-PAGE followed by fluorography. D) Auto-ubiquitylation of in vitro translated E6AP. In vitro translated, radiolabeled E6AP was incubated under standard ubiquitylation conditions with increasing concentrations of Cu(I)•THPTA (4 µM-40 µM) as indicated. C_0 and C_I, control reactions at 4 °C and 30 °C, respectively, in the absence of Cu(I)•THPTA. Reaction products were analyzed by SDS-PAGE followed by fluorography. The running position of non-modified E6AP is indicated by asterisk.

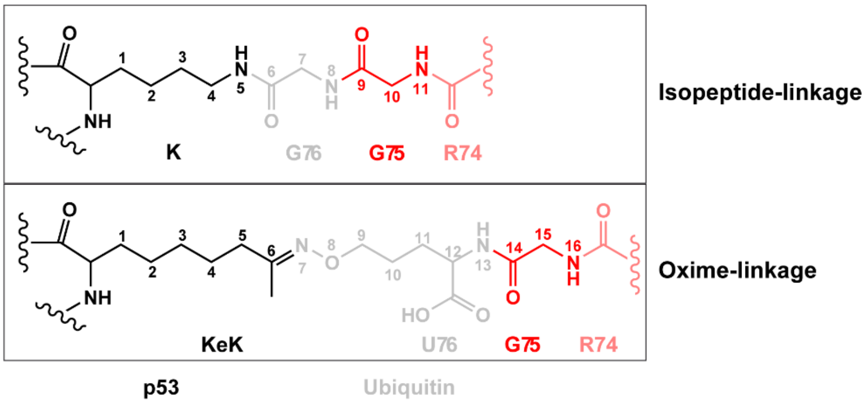

Figure S2. Comparison of the native isopeptide linkage between a lysine residue of p53 and the C-terminal glycine of ubiquitin and the oxime linkage between KeK and Ub76ONH$_2$**.**

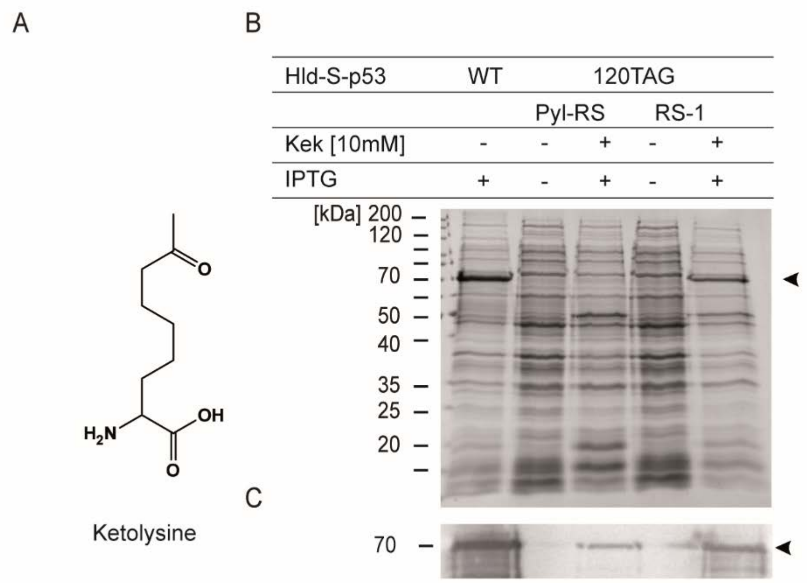

Figure S3. Incorporation of Ketolysine (KeK) into p53 at position 120. A) Structure of the unnatural amino acid Ketolysine (KeK). B) Co-expression of His_9-lipoyl (Hld) and Strep (S) tagged p53-120TAG, Pyl-$tRNA_{CUA}$ and Pyl-tRNA synthetase (Pyl-RS) of *M. barkeri* or a mutated form of it (RS-1; harboring the mutations L270I, Y271F, L274A, C313F, 266V) in *E. coli* BL21(DE3) in presence or absence of 10 mM KeK. Upon induction, aliquots were subjected to SDS-PAGE followed by Coomassie brilliant blue staining (upper panel) or by Western blot analysis against p53 (lower panel). Recombinantly expressed wild-type Hld-S-p53 (WT) was loaded as control. Running position of Hld-p53 is marked by an arrowhead.

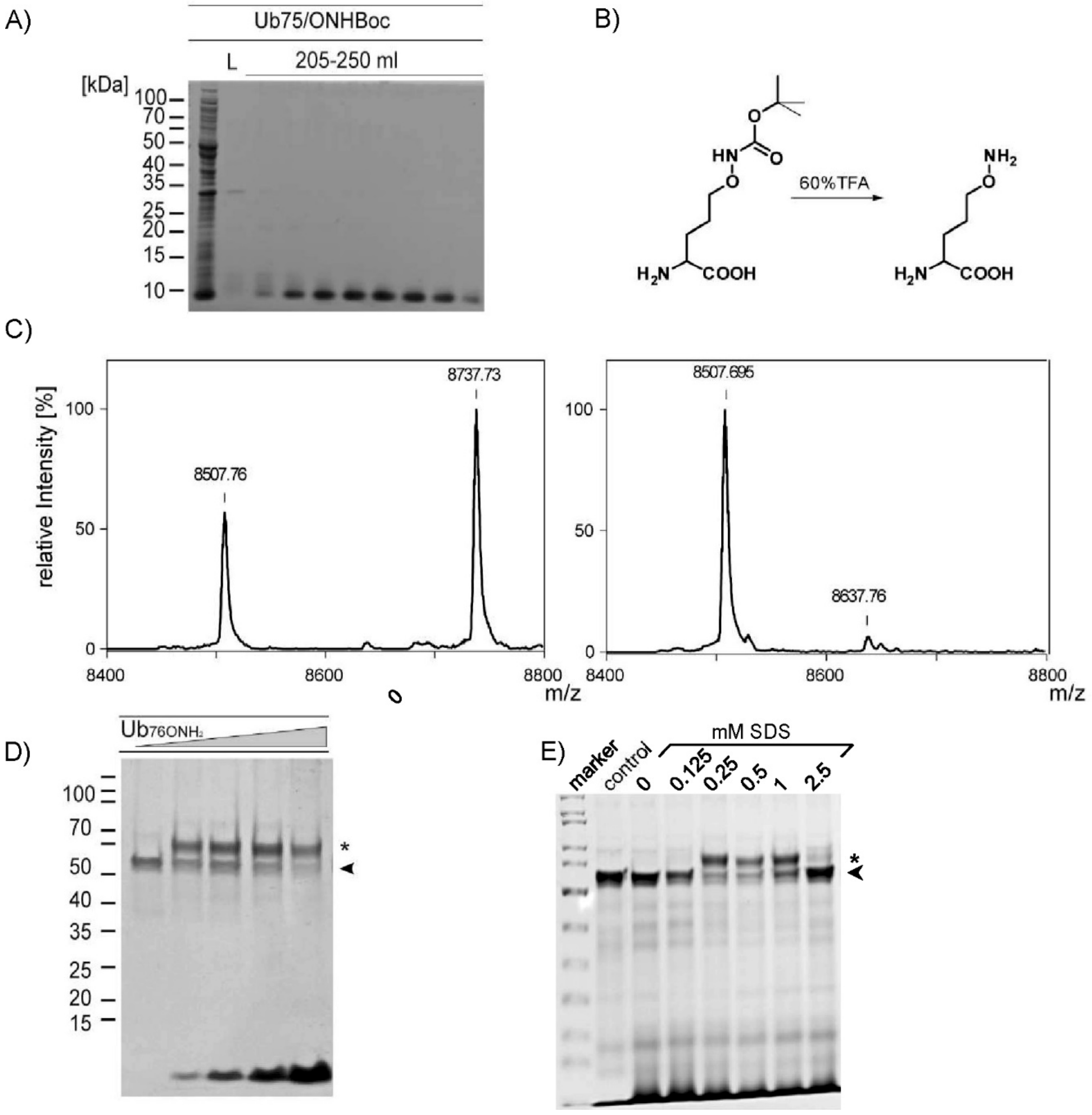

Figure S4. Generation of mono-functionalized Ub76ONH-Boc using ACS. A) Ub76ONH-Boc was generated and purified by size exclusion chromatography as described in the Experimental Section. An aliquot of the respective fractions was analyzed by SDS-PAGE followed by Coomassie blue staining. Note that incorporation of U1 did not proceed to completion (see panel C), resulting in a mixture of truncated ubiquitin (Ub75) and full-length Ub76ONH-Boc (Ub75/ONHBoc). B) Schematic of the deprotection of Nε-aminooxy-(tert-butoxycarbonyl)-L-lysine (U1) to Nε-aminooxy-L-lysine using 60 % TFA. C) Deconvoluted ESI-MS spectra of purified protected Ub75/Ub76ONH-Boc (left panel) and unprotected Ub75/Ub76ONH$_2$ (right panel). Calculated Mass: Ub75 [M+] = 8507.8 Da, Ub76ONHBoc [M+] = 8738.9 Da, Ub76-ONH$_2$ [M+] = 8637.8 Da. D) Efficiency of oxime ligation is slightly enhanced by increasing amounts of Ub76ONH$_2$. p53-120KeK (20 pmol) was mixed with 0.5 mM SDS and different molar excess (1:6.25-1:100) of the Ub76ONH$_2$/Ub75 mixture (see right panel in C). Samples were subjected to two freeze-thaw cycles, centrifuged, and subjected to SDS-PAGE followed by Coomassie blue staining. E) Generation of p53-120-Ub by oxime ligation at room temperature. p53-120KeK (20 pmol) was mixed with a 50-molar excess of Ub76ONH$_2$ in presence of increasing concentrations of SDS as indicated and incubated at 25 °C for 20 h. Reaction products were analyzed by SDS-PAGE followed by Coomassie blue staining. Running positions of p53-120KeK and p53-120-Ub are indicated by an arrow and an asterisk, respectively.

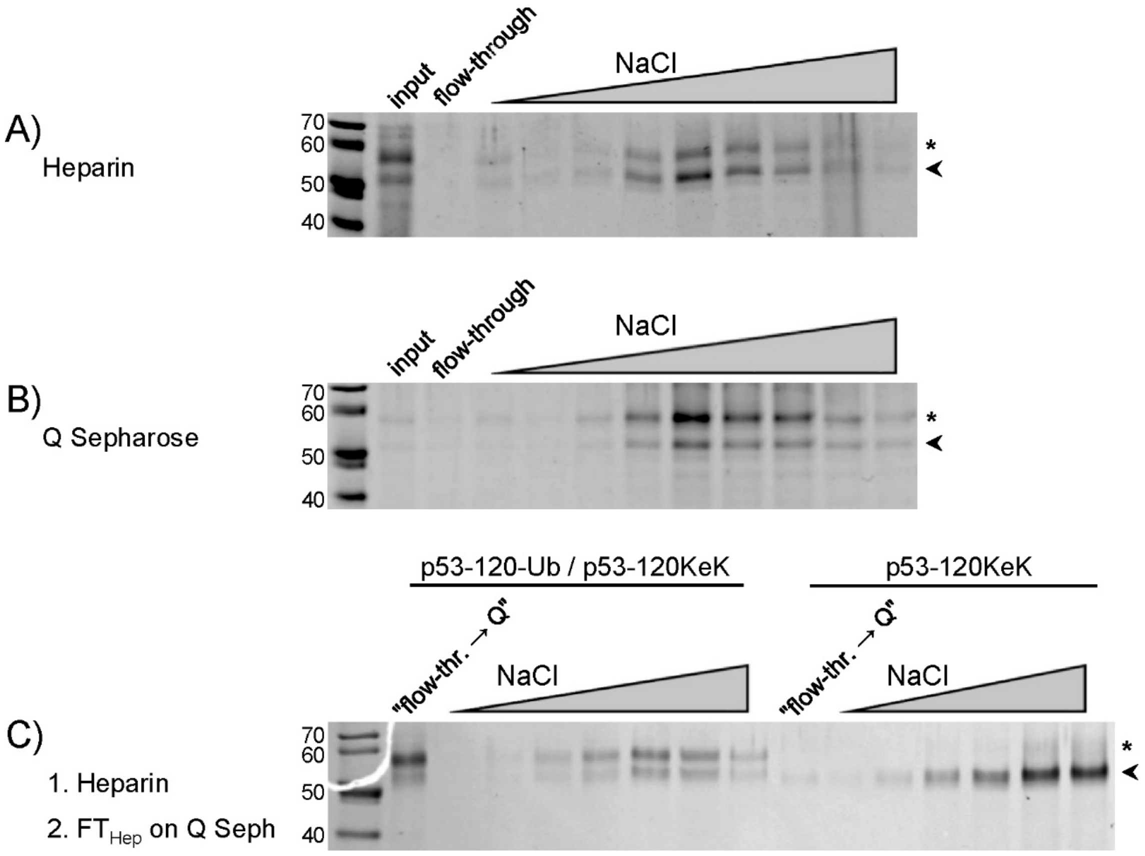

Fig. S5. Purification of p53-120-Ub via heparin affinity chromatography followed by anion exchange chromatography. A) Upon oxime ligation, the resulting mixture of p53-120-Ub / p53-120KeK was subjected to a heparin column and bound proteins were eluted by a salt gradient from 150 mM to 1 M NaCl. The p53 variants eluted between 280-350 mM NaCl. However, compared to the input (20 %), the ratio between p53-120-Ub to p53-120KeK was reversed. This indicates that p53-120-Ub has a lower affinity to heparin than p53-120KeK and that approximately 50 % of p53-120-Ub did not bind to heparin under the conditions used. B) Upon oxime ligation, the resulting mixture of p53-120-Ub / p53-120KeK was subjected to a Q sepharose column and bound proteins were eluted by a salt gradient from 150 mM to 1 M NaCl. The majority of the p53 variants eluted between 350-400 mM NaCl, and there was no difference in the elution behavior between p53-120-Ub and p53-120KeK. Input represents 2 % of the mixture applied. C) Because of the different binding behavior of p53-120-Ub and p53-120-KeK, upon oxime ligation the mixture of p53 variants was loaded first onto a heparin column. The flow-through (FT) was collected and applied to Q sepharose. Proteins bound to Q sepharose were eluted with 400 mM NaCl ("flow-thr. → Q"). As control, p53-120KeK was subjected to the same purification procedure, showing that in contrast to p53-120-Ub, p53-120KeK bound to heparin almost quantitatively and eluted between 280-350 mM NaCl. 5 % of the respective elution fractions were subjected to SDS-PAGE followed by Coomassie blue staining. Running positions of p53-120KeK and p53-120-Ub are indicated by an arrow and an asterisk, respectively.

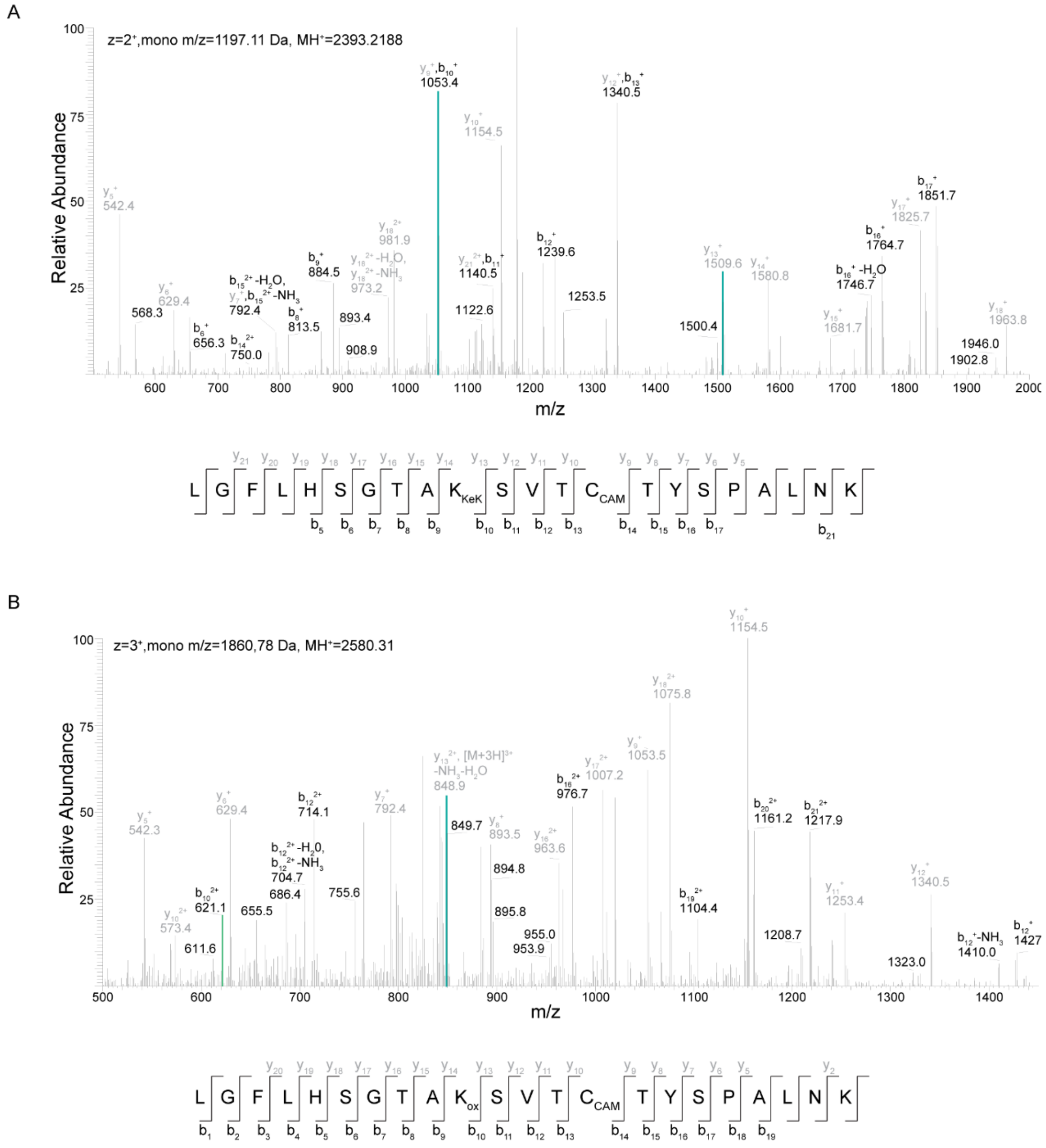

Figure S6. Analysis of trypsin-digested p53-120KeK and p53-120-Ub by mass spectrometry. A) LC-MS/MS spectrum of the p53 peptide comprising amino acids 111-132 with KeK at position 120 (K_{KeK}). B) LC-MS/MS spectrum of the oxime-linked p53 peptide comprising amino acids 111-132 with Ub attached at position 120 (K_{ox}). Note that upon trypsin digest, KeK at position 120 of p53 carries G75 and the condensated U1 at position 76 of Ub76ONH$_2$ (as trypsin cleaves after R74 of ubiquitin). KeK, Ketolysine; CAM, Carbamidomethyl.

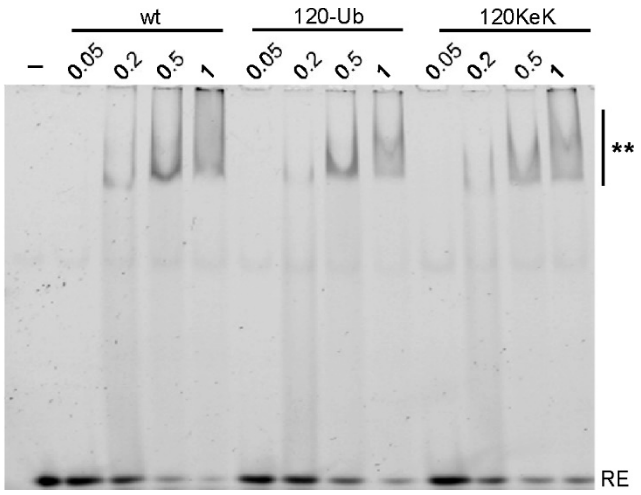

Fig. S7. Characterization of the DNA binding ability of p53 variants by EMSA. Increasing concentrations of p53-120-Ub, p53-120KeK, and wild-type (wt) p53 were incubated with 10 nM fluorescein-labeled p21 response element (RE) and analyzed on a 4% native TBE polyacrylamide gel followed by fluorescence read out at 473 nm. -, control reaction in the absence of p53; RE, running position of the unbound RE; **, running position of the RE-p53 complex.

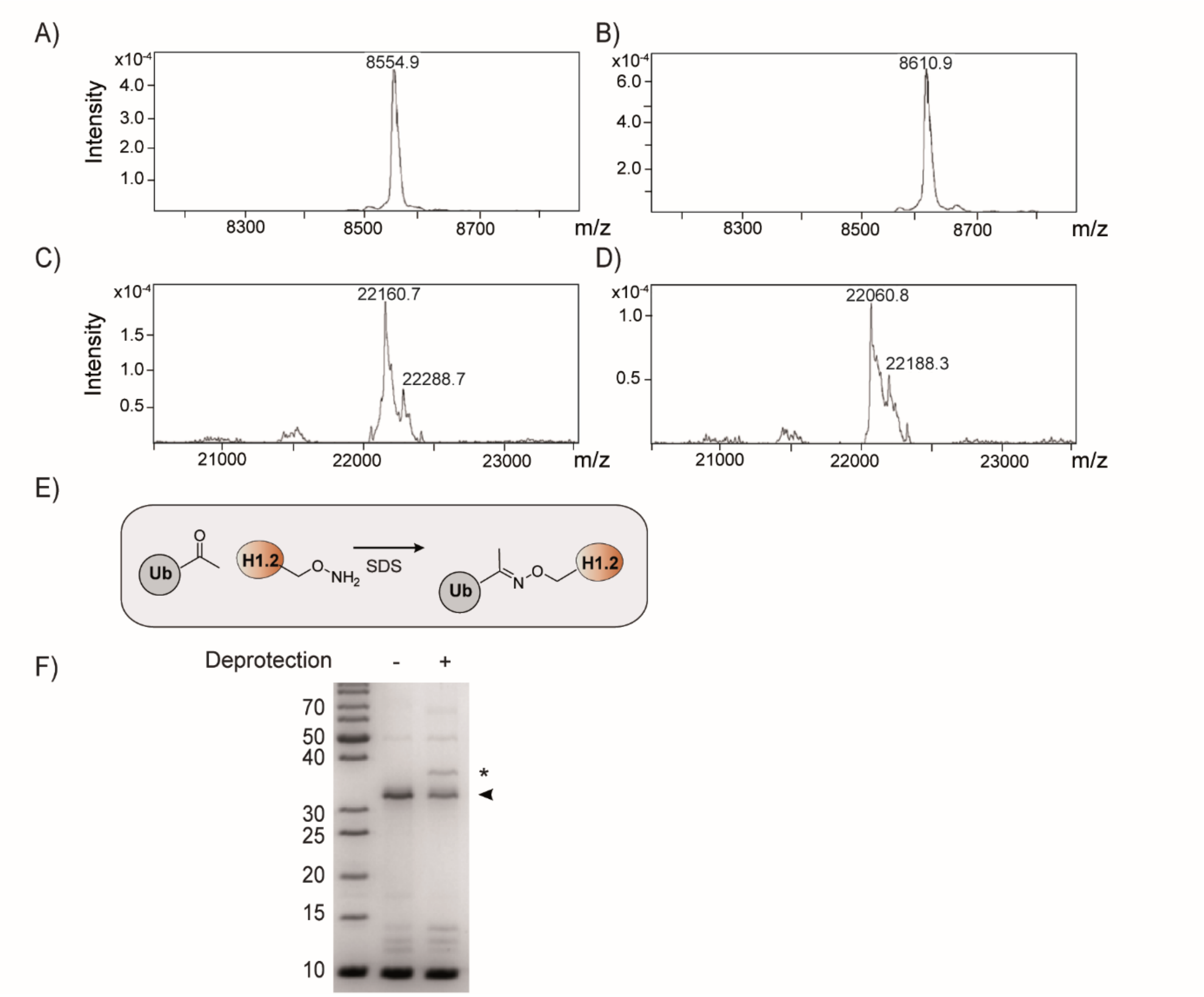

Figure S8. Generation of mono-ubiquitylated H1.2 by oxime ligation. Deconvoluted ESI-MS spectra of A) Ub-75C, calculated mass [M+H+] = 8553.9 Da and measured mass [M+H+] = 8554.9 Da; (B) mono-functionalized Ub-75C-Keto, calculated mass [M+H+] = 8610.0 Da and measured mass [M+H+] = 6610.9 Da; C) H1.2 mono-functionalized at position 206 (H1.2-206U1), calculated mass [M+H+] = 22289.7 Da and [M+H+-Met] = 22185.5 Da and measured mass [M+H+] = 22289.7Da and [M+H+-Met] = 22160.7 Da; D) H1.2-206-aminooxy, calculated mass [M+H+] = 22189.7 Da and [M+H+-Met] = 22058.5 Da and measured mass [M+H+] = 22188.3 Da and [M+H+-Met] = 22060.8 Da. E) Schematic of the generation of mono-ubiquitylated H1.2 by oxime ligation. F) Analysis of oxime ligation between Ub-75C-Keto and untreated (protected) H1.2 206U1 (-) or TFA-treated (deprotected) H1.2-206U1 (+) by SDS-PAGE followed by Coomassie blue staining. TFA treatment was performed with 67 % TFA for 2 h at 23 °C. Subsequently, oxime ligation was performed using 50 µM Ub75C-Keto and 10 µM H1.2-206U1 with 2 mM SDS at pH 7.0 and 3 freeze-thaw cycles. Running positions of molecular mass markers, unmodified H1.2-206U1 (arrow), and mono-ubiquitylated H1.2 (asterisk) are indicated.

6. Supporting References

[1] a) G. A. Rosenthal, D. L. Dahlman, P. A. Crooks, S. N. Phuket, L. S. Trifonov, *J. Agr. Food Chem.* **1995**, *43*, 2728-2734; b) F. Liu, J. Thomas, T. R. Burke, Jr., *Synthesis (Stuttg)* **2008**, *15*, 2432-2438.

[2] Y. Huang, W. Wan, W. K. Russell, P. J. Pai, Z. Wang, D. H. Russell, W. Liu, *Bioorg. Med. Chem. Lett.* **2010**, *20*, 878-880.

[3] S. Eger, M. Scheffner, A. Marx, M. Rubini, *J. Am. Chem. Soc.* **2010**, *132*, 16337-16339.

[4] D. Schneider, T. Schneider, D. Rösner, M. Scheffner, A. Marx, *Bioorg. Med. Chem.* **2013**, *21*, 3430-3435.

[5] M. Stanley, S. Virdee, *ChemBioChem* **2016**, *17*, 1472-1480.

[6] A. N. Bullock, J. Henckel, B. S. DeDecker, C. M. Johnson, P. V. Nikolova, M. R. Proctor, D. P. Lane, A. R. Fersht, *Proc. Natl. Acad. Sci. U S A* **1997**, *94*, 14338-14342.

[7] a) H. Neumann, S. M. Hancock, R. Buning, A. Routh, L. Chapman, J. Somers, T. Owen-Hughes, J. van Noort, D. Rhodes, J. W. Chin, *Mol. Cell* **2009**, *36*, 153-163; b) H. Neumann, S. Y. Peak-Chew, J. W. Chin, *Nat. Chem. Biol.* **2008**, *4*, 232-234.

[8] F. Offensperger, F. Muller, J. Jansen, D. Hammler, K. H. Gotz, A. Marx, C. L. Sirois, S. J. Chamberlain, F. Stengel, M. Scheffner, *Cell Chem. Biol.* **2020**, *27*, 1510-1520 e1516.
